# How sleeping minds decide: State-specific reconfigurations of lexical decision-making

**DOI:** 10.1371/journal.pcbi.1014007

**Published:** 2026-02-23

**Authors:** Tao Xia, Chuan-Peng Hu, Başak Türker, Esteban Munoz Musat, Lionel Naccache, Isabelle Arnulf, Delphine Oudiette, Xiaoqing Hu

**Affiliations:** 1 State Key Laboratory of Cognitive Science and Mental Health, Institute of Psychology, Chinese Academy of Sciences, Beijing, China; 2 Department of Psychology, The University of Hong Kong, Hong Kong SAR, China; 3 School of Psychology, Nanjing Normal University, Nanjing, China; 4 Sorbonne Université, Institut du Cerveau—Paris Brain Institute—ICM, INSERM, CNRS, Paris, France; 5 AP-HP, Hôpital Pitié-Salpêtrière, Service de Neurophysiologie Clinique, Paris, France; 6 AP-HP, Hôpital Pitié-Salpêtrière, Service des Pathologies du Sommeil, National Reference Centre for Narcolepsy, Paris, France; 7 HKU-Shenzhen Institute of Research and Innovation, Shenzhen, China; Pantheon-Sorbonne University: Universite Paris 1 Pantheon-Sorbonne, FRANCE

## Abstract

Sleep has traditionally been conceptualized as a state of cognitive disconnection, yet emerging evidence indicates that decision-making capacities persist across sleep stages. Here, we elucidate the computational mechanisms underlying real-time lexical decision-making during polysomnographically-verified sleep, using facial electromyography and hierarchical drift diffusion modeling in both healthy individuals and participants with narcolepsy. We found that lexical decision-making was preserved during N1 and lucid REM sleep, but relied on distinct computational strategies: in N1 sleep, both enhanced sensory-motor processing and increased evidence accumulation supported decisions about words, whereas in lucid REM sleep, lexical decisions were driven exclusively by evidence accumulation processes. Cross-state comparisons revealed two fundamental principles: (1) Selective preservation—during N1 sleep, lexical decisions for words were maintained while those for pseudowords were selectively impaired, indicating that cognitive resources during sleep are preferentially allocated to meaningful stimuli; (2) Parallel strategic adaptations—during lucid REM sleep, participants increased their decision thresholds, requiring more evidence before responding, which helped maintain accuracy even though the efficiency of evidence accumulation was reduced. Our findings demonstrate that, rather than a passive decline, sleep involves dynamic and state-specific reconfiguration of the computational mechanisms underlying decision-making, with important implications for understanding consciousness and cognitive flexibility.

## Author summary

Sleep is traditionally viewed as a state of cognitive disconnection, where the mind cannot interact with the world. We challenged this view by investigating how the sleeping brain makes decisions. We asked participants, including frequent lucid dreamers, to distinguish between real words and nonsense words while asleep, responding via subtle facial muscle movements. We found that the sleeping mind does not simply shut down; instead, it fundamentally changes how it processes information. During light sleep, participants retained the ability to respond quickly and accurately to familiar words but stopped responding to nonsense words, suggesting the brain prioritizes meaningful content. In lucid REM sleep—where dreamers are aware they are dreaming—behavior shifted differently: responses became much slower. Our mathematical modeling revealed that because the brain was less efficient at gathering information in this state, it adopted a “conservative” strategy, requiring more certainty before acting to maintain accuracy. These findings demonstrate that sleep is not a passive state, but a dynamic one where the brain actively reconfigures its decision-making strategies to suit the distinct states of consciousness.

## Introduction

Sleep has traditionally been viewed as a state of behavioral unresponsiveness and cognitive disconnection from the environment, characterized by the wholesale suspension of complex cognitive functions [1]. This classical perspective, rooted in early behavioral observations of reduced responsiveness, has profoundly shaped our understanding of consciousness-cognition relationships and the functional architecture of the sleeping brain [2]. However, accumulating evidence now challenges this perspective, revealing that the sleeping brain can continue to process sensory input, form new associations, and—under specific conditions—even engage in goal-directed behaviors [3–8]. These findings suggest that cognitive processing during sleep is more flexible and adaptive than previously thought, capable of systematic reorganization rather than uniform decline across different states of consciousness.

Recent advances highlight this flexibility across multiple domains. For example, individuals can communicate in real time during lucid dreams [5], acquire new word associations during non-rapid eye movement (NREM) sleep [7,9], and perform complex tasks such as lexical decision-making using volitional facial muscle contractions [6]. Despite these advances, a critical gap remains: behavioral data alone cannot reveal how underlying cognitive processes adapt across states of consciousness. Measures such as accuracy and response time can be insufficient to distinguish between different cognitive strategies, as similar behavioral outcomes may arise from distinct underlying processes or compensatory adaptations to altered brain states [10,11]. This challenge is magnified in sleep, where the cognitive mechanisms supporting overt behavior remain poorly understood. To disentangle these possibilities, computational modeling—specifically, drift diffusion modeling (DDM)—provides a powerful approach by decomposing observed behavior into distinct components: evidence accumulation (drift rate, indexing the efficiency of information integration), sensory-motor processing (non-decision time, representing stimulus encoding and response preparation), decision thresholds (the amount of evidence required before making a choice, reflecting response caution), and starting point bias (pre-decisional tendencies) [11,12]. This framework has demonstrated that various neurological and pharmacological states can selectively affect these decision components, underscoring that decision-making is modular and differentially sensitive to changes in brain state [11,13].

Adapting computational modeling to sleep necessitates accounting for the unique neurophysiological characteristics of each sleep stage, which may require distinct mechanistic interpretations of cognitive processes. N1 sleep, for example, retains some thalamocortical connectivity and limited cortical integration but shows reduced attentional control [3,14], raising the possibility that automatic processing is preserved while controlled operations are compromised. In N2 sleep, further reductions in cortical connectivity, the presence of sleep spindles and K-complexes, and diminished sensory responsiveness may preclude complex decision-making altogether [15,16]. REM sleep introduces a different neural context: heightened cortical activation occurs alongside altered neurotransmitter dynamics and disrupted sensory-motor coupling [17–19]. Lucid REM sleep is particularly unique, as it allows for metacognitive awareness within this altered neurochemical environment, potentially supporting explicit cognitive control strategies not present in other stages [20,21].

To operationalize these hypotheses, we employ the lexical decision-making paradigm, which robustly engages both automatic and controlled cognitive processes [22]. Lexical decisions for familiar words typically rely on rapid access to well-established semantic representations and are considered relatively automatic, whereas lexical decisions for pseudowords demand controlled phonological analysis and the evaluation of novel linguistic input [23,24]. This distinction enables a critical test: if decision-making during sleep is adaptively reorganized rather than uniformly degraded, we should observe preserved automatic lexical decisions for familiar words, with selective impairment in decisions involving novel stimuli such as pseudowords. Conversely, if decision-making is uniformly degraded across sleep, both word and pseudoword decisions should show similar declines in performance, reflecting a generalized disruption of cognitive function regardless of stimulus type.

The outcomes of such a test have implications that extend well beyond the lexical domain. If decision-making mechanisms are indeed systematically reconfigured across sleep stages, this challenges prevailing theories that conceptualize sleep merely as a state of diminished consciousness or reduced cognitive capacity [18,25]. Instead, such findings would support the view that each state of consciousness presents a distinct computational environment, necessitating adaptive changes in cognitive processes [26]. This perspective reframes altered states of consciousness from periods of passive dysfunction to opportunities for active reorganization, potentially serving critical functions such as memory consolidation and synaptic plasticity [27]. Apparent impairments in cognition during sleep may therefore reflect not simple deficits, but rather adaptive reconfigurations that optimize cognitive processing for the unique demands of each state. Adopting this framework encourages a shift in research focus—from merely assessing whether cognitive processes persist during sleep, to elucidating how they are dynamically restructured across different states of consciousness.

In this study, we combine real-time behavioral measurement using facial electromyography with hierarchical drift diffusion modeling to systematically characterize how lexical decision-making adapts across wakefulness, N1 sleep, and lucid REM sleep. To reliably access lucid REM sleep—a state that is otherwise rare in the general population—we included participants with narcolepsy, who frequently experience this phenomenon [28]. This methodological approach enables direct, within-subject comparisons of decision-making across distinct states of consciousness. By decomposing lexical decisions into their constituent computational components, our analyses distinguish between global impairments and state-specific adaptive strategies, offering novel insights into the mechanisms by which decision-making is reorganized during altered states of consciousness.

## Results

To examine how decision-making processes adapt across states of consciousness, we applied hierarchical drift diffusion modeling (HDDM) to lexical decision performance during wakefulness, N1 sleep, N2 sleep, REM sleep, and lucid REM sleep. This approach decomposed performance into sensory-motor processing (non-decision time), evidence accumulation (drift rate), and response caution (decision threshold). We first compared several candidate HDDM specifications and selected a full model in which all three parameters varied as functions of stimulus type, sleep stage, and their interaction, as this model provided a substantially better fit than simpler alternatives (see S5 Table and Methods: Model Comparison).

Our analytical approach was stratified by group: comprehensive computational modeling across all sleep stages was performed for narcolepsy participants (who contributed sufficient trials; S2 Table), while healthy participants served as a behavioral validation cohort for N1 sleep findings. Results are reported on both original measurement scales (percentages, seconds) and statistically-analyzed scales (logit, log), with 95% highest density intervals (HDI) characterizing posterior uncertainty.

### Wakefulness establishes distinct decision-making profiles

Wakefulness revealed distinct lexical decision-making profiles between healthy participants and individuals with narcolepsy, establishing critical baselines for interpreting sleep-related adaptations.

**Fig 1:**
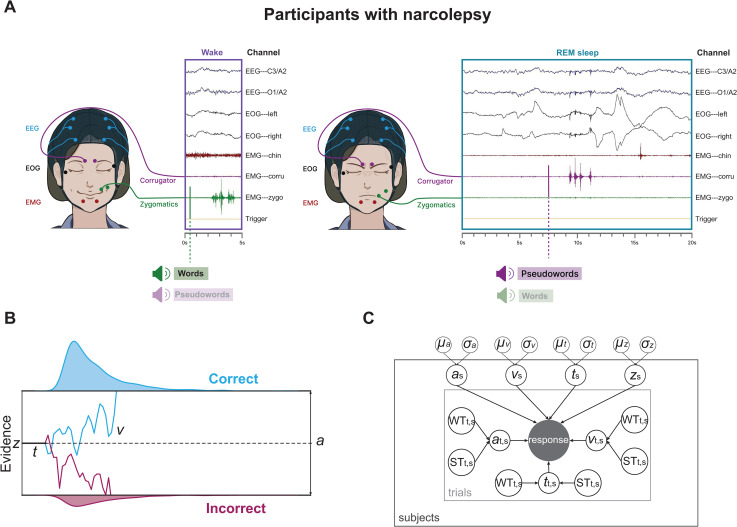
Experiment design and computational modeling framework. (A). Experimental Procedure. Participants performed a lexical decision task during daytime nap sessions, responding to spoken stimuli (words or pseudowords) via volitional facial muscle contractions. Responses were mapped to either frowning (corrugator muscle) or smiling (zygomatic muscle), counterbalanced across participants. Inset traces show example EMG responses during wakefulness and Lucid REM sleep. Stimuli were presented in a pseudorandomized order to prevent repetition. (B) Drift Diffusion Model (DDM) Schematic. The DDM decomposes decision-making into distinct cognitive components: the drift rate (***v***); the decision threshold (***a***); the non-decision time (***t***); and the starting point (***z***); The schematic illustrates the noisy accumulation of evidence over time until a boundary is reached. (C) Hierarchical Bayesian HDDM Framework. The model estimates parameters simultaneously at the group and individual levels. Group-level distributions (mean ***µ***, variance ***σ***) constrain individual parameters (***z***, ***a***, ***v***, ***t***), allowing the model to leverage shared information across participants. At the Trial level (T), parameters ***v***, ***a***, and ***t*** vary as functions of Word Type (WT) and Sleep Stage (ST). Shaded nodes represent observed data (Accuracy, RT); unshaded nodes represent latent parameters.

**Fig 2:**
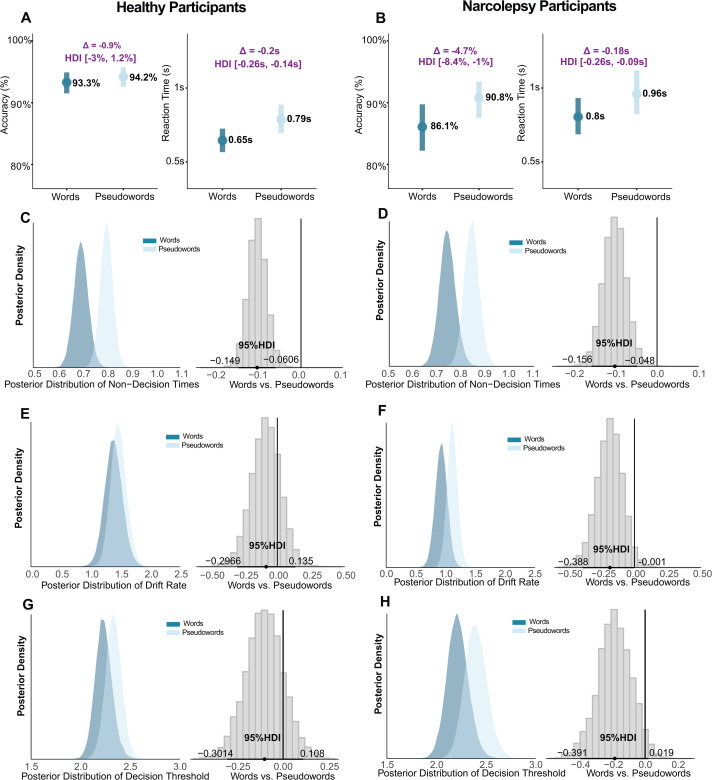
Behavioral and computational profiles of lexical decision-making during wakefulness. (A, B) Behavioral Results: Response Accuracy and Reaction Time. Bayesian Linear Mixed Model (BLMM) results for lexical decisions in HP (left panels) and NP (right panels) during wakefulness. The x-axis represents the estimated mean for response accuracy (top) and reaction time (bottom). Light blue lines indicate the 95% Highest Density Interval (HDI) for pseudowords, and blue lines indicate words. (C–H) Posterior distributions of HDDM parameters for Healthy Participants (HP; left columns, panels C, E, G) and Participants with Narcolepsy (NP; right columns, panels D, F, H). For each parameter, the left sub-panel shows the posterior estimates for words (blue) and pseudowords (light blue), while the right sub-panel displays the posterior distribution of the difference (contrast) between them. Horizontal black lines indicate the 95% Highest Density Interval (HDI). A difference is considered statistically credible if the 95% HDI of the contrast excludes zero (vertical gray line).

In narcolepsy participants, lexical decisions exhibited a speed-accuracy dissociation. Words elicited faster reaction times than pseudowords (0.80s vs. 0.96s, difference: -0.18s, 95% HDI: [-0.26s, -0.09s]; log scale: -0.176, 95% HDI: [-0.260, -0.095], Fig 2B right), yet showed lower accuracy (86.1% vs. 90.8%, difference: -4.7%, 95% HDI: [-8.4%, -1.0%]; log scale: -0.460, 95% HDI: [-0.829, -0.101], Fig 1B left)—both credible intervals excluding zero. Drift diffusion modeling revealed that while words benefited from faster sensory-motor processing (non-decision time: -0.101s, 95% HDI: [-0.156, -0.048], Fig 2D), they suffered from impaired evidence accumulation (drift rate: -0.184, 95% HDI: [-0.388, -0.001], Fig 2F), consistent with cognitive impairments in narcolepsy [29]. Decision thresholds showed no reliable differences across stimulus types in both groups (all 95% HDIs overlapped with 0, Fig 2H).

Healthy participants demonstrated a different profile, with efficient lexical processing characterized by faster reaction times for words than pseudowords (0.65s vs. 0.79s, difference: -0.2s, 95% HDI: [-0.26s, -0.14s]; log scale: -0.199, 95% HDI: [-0.257, -0.141], Fig 2A right), driven by more efficient sensory-motor processing (non-decision time difference: -0.106s, 95% HDI: [-0.149, -0.061], Fig 2C). Accuracy was comparable between stimulus types (93.3% vs. 94.2%, difference: -0.9%, 95% HDI: [-3.0%, 1.2%]; logit scale: -0.156, 95% HDI: [-0.516, 0.195], Fig 1A left), with credible intervals overlapping zero, consistent with equivalent evidence accumulation and decision threshold (all 95% HDIs overlapped with 0, Fig 2E and 2G).

These contrasting computational profiles—efficient sensory-motor processing with intact evidence accumulation in healthy participants versus preserved sensory-motor efficiency coupled with impaired evidence accumulation in narcolepsy—establish critical baselines for distinguishing genuine sleep-related adaptations from pre-existing group differences.

### State-dependent mechanisms support lexical decisions during N1 and lucid REM sleep

Despite transitions into sleep, participants continued to distinguish words from pseudowords during both N1 sleep and lucid REM sleep, though through state-specific computational mechanisms.

**Fig 3:**
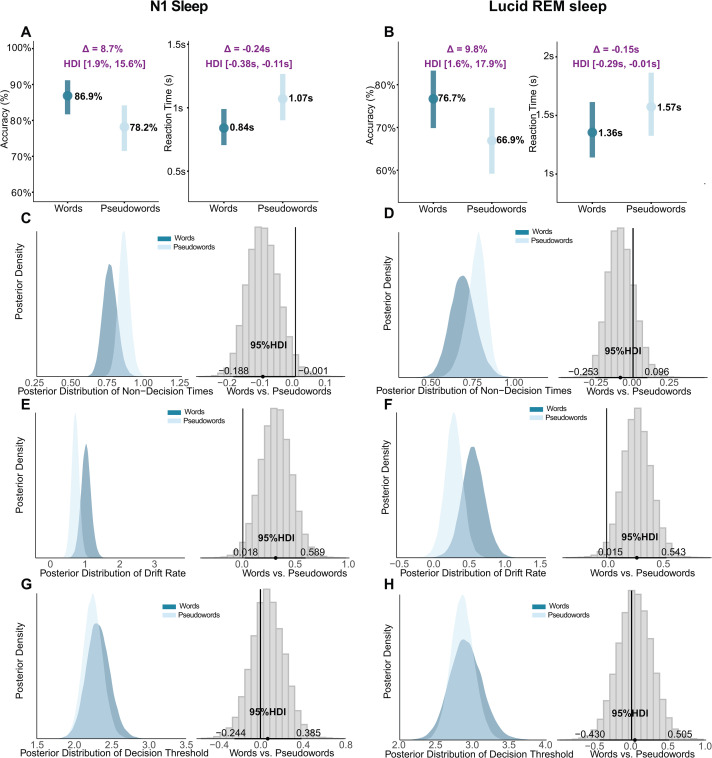
Mechanism of lexical decision during N1 and lucid REM sleep. (A, B) BLMM results for response accuracy and reaction time in lexical decisions during N1 sleep and lucid REM sleep in participants with narcolepsy. The N1 sleep results for healthy participants exhibited the same behavioral pattern and computational mechanisms as in participants with narcolepsy and are presented in S2 Fig. The y-axis represents the estimated mean response accuracy or reaction time. The light blue line indicates the 95% Highest Density Interval (HDI) of the posterior probability for pseudowords, while the blue line represents words. (C–H) HDDM Parameters during N1 and Lucid REM Sleep. Posterior distributions for N1 sleep (panels C, E, G) and Lucid REM sleep (panels D, F, H) in participants with narcolepsy. Each panel displays parameter estimates for words (blue) vs. pseudowords (light blue) alongside their contrast distribution. Horizontal black lines mark the 95% HDI; contrasts excluding zero (gray line) indicate credible differences. Parameters shown are: (C, D) Non-decision time (***t***); (E, F) Drift rate (***v***); and (G, H) Decision threshold (***a***).

During N1 sleep, both groups exhibited robust word advantages. Narcolepsy participants exhibited robust word advantages, with faster reaction times (0.84s vs. 1.07s, difference: -0.24s, 95% HDI: [-0.38s, -0.11s]; log scale: -0.241, 95% HDI: [-0.381, -0.105], Fig 3A right) and higher accuracy for words (86.9% vs. 78.2%, difference: 8.7%, 95% HDI: [1.9%, 15.6%]; log scale: 0.615, 95% HDI: [0.136, 1.093], Fig 3A left), with credible intervals excluding zero. HDDM analyses revealed dual mechanisms: enhanced sensory-motor processing (non-decision time difference: -0.098s, 95% HDI: [-0.188, -0.001], Fig 3C) and more efficient evidence accumulation (drift rate difference: 0.308, 95% HDI: [0.018, 0.589], Fig 3E). Decision thresholds remained stable (95% HDI overlapped with 0, Fig 3G).

Healthy participants showed a similar pattern, with faster reaction times (0.63s vs. 0.98s, difference: -0.45s, 95% HDI: [-0.67s, -0.23s]; log scale: -0.450, 95% HDI: [-0.673, -0.233], S2A Fig right) and higher accuracy for words (94.6% vs. 75.8%, difference: 18.6%, 95% HDI: [7.8%, 31.6%]; log scale: 1.738, 95% HDI: [0.673, 2.930], S2A Fig left), supported by the same dual mechanisms (non-decision time: -0.213s, 95% HDI: [-0.367, -0.062]; drift rate: 0.732, 95% HDI: [0.105, 1.433], S2B, S2C and S2D Fig). This cross-group convergence demonstrates that N1 sleep selectively preserves automatic semantic processing while compromising controlled evaluation of novel linguistic material.

In lucid REM sleep, participants with narcolepsy maintained the capacity for lexical decision-making but employed a computationally distinct strategy. Behaviorally, words continued to show advantages in both reaction time (1.36s vs. 1.57s, difference: -0.15s, 95% HDI: [-0.29s, -0.01s]; log scale: -0.149, 95% HDI: [-0.291, -0.011], Fig 3B right) and accuracy (76.7% vs. 66.9%, difference: 9.8%, 95% HDI: [1.6%, 17.9%]; log scale: 0.492, 95% HDI: [0.080, 0.908], Fig 3B left), with both credible intervals excluding zero. However, HDDM revealed a fundamental shift in the underlying mechanisms: the word advantage in lucid REM was driven exclusively by enhanced evidence accumulation (median_*diff*_ = 0.274, 95% HDI [0.015, 0.543], Fig 3F), with no detectable differences in sensory-motor processing (non-decision times) or response caution (decision thresholds) between stimulus types (all 95% HDIs overlapped with 0, Fig 3 D and Fig 3H). This contrasts sharply with N1 sleep, where both sensory-motor and evidence accumulation processes contributed to word recognition advantages.

These findings demonstrate that lexical decision-making persists during sleep through state-specific computational reconfigurations rather than uniform continuation of waking processes. N1 sleep preserves both the sensory-motor and evidence accumulation advantages characteristic of automatic word recognition, suggesting that core semantic networks remain accessible despite reduced arousal. Lucid REM sleep, in contrast, relies exclusively on enhanced evidence accumulation, indicating that while post-sensory decision processes remain functional, the sensory-motor pathways supporting rapid word recognition are compromised or decoupled. This dissociation reveals that different sleep states selectively preserve or impair distinct components of the decision-making architecture, challenging models that treat sleep as a state of global cognitive decline.

### Adaptive reconfigurations of decision-making across wakefulness, N1 sleep, and lucid REM sleep

Having established that lexical decision-making persists during sleep through state-specific mechanisms, we next examined how computational parameters change as participants transition between states of consciousness. Comparisons across states of consciousness revealed systematic adaptations in decision-making, rather than uniform degradation, with distinct effects for familiar words versus novel pseudowords.

In narcolepsy participants, lexical decisions for words remained stable during N1 sleep compared to wakefulness. Reaction times (log scale: -0.043, 95% HDI: [-0.163, 0.073]) and accuracy (log scale: -0.069, 95% HDI: [-0.534, 0.377]) showed no reliable differences, with credible intervals overlapping zero. Drift diffusion parameters (***t***, ***v***, ***a***) similarly showed no reliable changes (all 95% HDIs overlapped with 0). In contrast, pseudoword decisions were selectively impaired: accuracy declined (log scale: 1.008, 95% HDI: [0.541, 1.432]), driven computationally by reduced evidence accumulation (drift rate: -0.468, 95% HDI: [-0.688, -0.252]), while reaction times and other parameters showed no reliable changes (all 95% HDI overlapped with zero).

**Fig 4:**
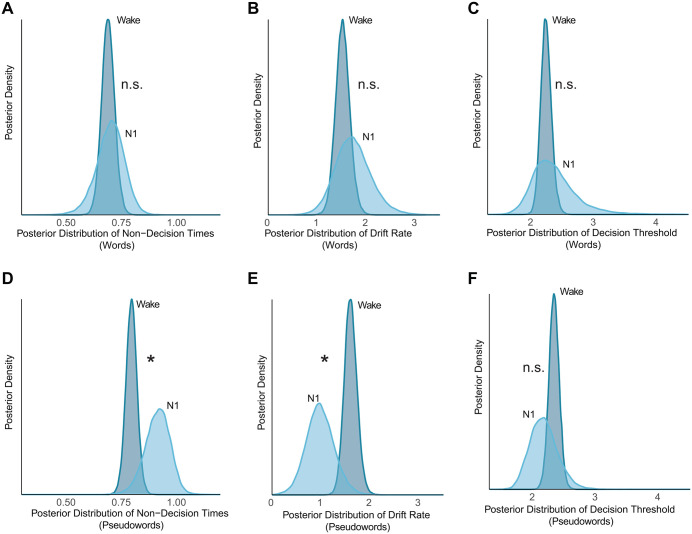
Comparison of Decision Parameters between Wakefulness and N1 Sleep (HP Group). Posterior distributions of HDDM parameters for words and pseudowords in the Healthy Participants. (Note: The NP group exhibited a qualitatively similar pattern of selective pseudoword impairment; see Results). Panels depict (A, D) non-decision time, (B, E) drift rate, and (C, F) decision threshold. Asterisks (*) denote credible differences, defined as the 95% Highest Density Interval (HDI) of the posterior contrast excluding zero. "n.s." indicates no reliable difference (95% HDI overlaps with zero).

Healthy participants replicated this selective preservation pattern. Words remained stable across wake and N1 sleep (RT: median_*diff*_ = 0.020, 95% HDI [-0.106, 0.148]; accuracy: median_*diff*_ = -0.163, 95% HDI [-1.216, 0.765]; Drift diffusion parameters (***t***, ***v***, ***a***): all 95% HDIs overlapped zero, Fig 4A, 4B and 4C), while pseudowords showed impairment (RT: median_*diff*_ = -0.199, 95% HDI [-0.345, -0.057]; accuracy: median_*diff*_ = 1.519, 95% HDI [0.823, 2.236]; both credible intervals excluded zero), driven by both delayed sensory-motor processing (non-decision time: -0.120s, 95% HDI: [-0.215, -0.009], Fig 4D) and reduced evidence accumulation (drift rate: 0.630, 95% HDI: [0.074, 1.115], Fig 4E). This cross-group convergence demonstrates that N1 sleep selectively preserves automatic semantic processing while compromising controlled evaluation of novel material.

In participants with narcolepsy, reaction times increased substantially for both stimulus types relative to wakefulness (words difference: -0.523, 95% HDI: [-0.646, -0.389]; pseudowords difference: -0.494, 95% HDI: [-0.622, -0.366]) and N1 sleep (words difference: -0.478, 95% HDI: [-0.627, -0.332]; pseudowords difference: -0.384, 95% HDI: [-0.537, -0.241]), with all credible intervals excluding zero. Accuracy declined for both stimulus types, but pseudowords showed substantially greater impairment: word accuracy decreased moderately relative to wakefulness (median_*diff*_: 0.631, 95% HDI: [0.207, 1.060]) and N1 sleep (median_*diff*_: 0.699, 95% HDI: [0.209, 1.215]), whereas pseudoword accuracy dropped dramatically relative to wakefulness (median_*diff*_: 1.582, 95% HDI: [1.143, 2.005]) and N1 sleep (median_*diff*_: 0.576, 95% HDI: [0.122, 1.016]), with all credible intervals excluding zero, representing approximately twice the decline magnitude.

**Fig 5:**
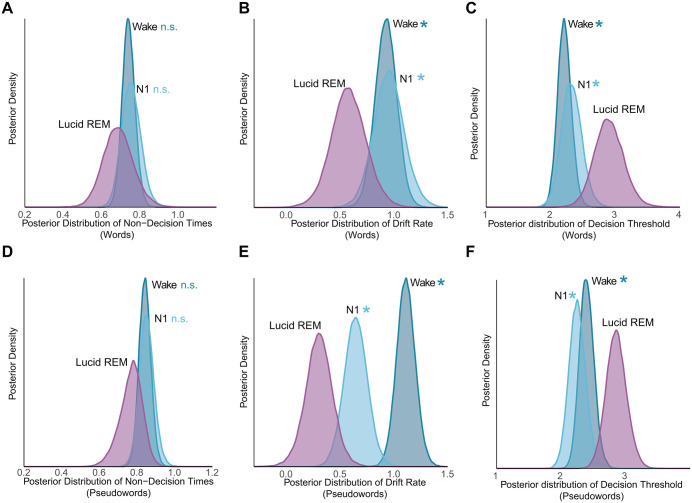
Slower and less efficient lexical decisions during lucid REM sleep. Posterior distributions of decision parameters for words and pseudowords in the NP group, comparing lucid REM sleep against Wakefulness, and N1 sleep. Parameters shown are: (A, D) non-decision time (***t***), (B, E) drift rate (***v***), and (C, F) decision threshold (***a***). Note that the direct comparison between Wakefulness and N1 is detailed in the Results section. Asterisks (*) denote credible differences, defined as the 95% Highest Density Interval (HDI) of the posterior contrast excluding zero. "n.s." indicates no reliable difference (95% HDI overlaps with zero).

Drift diffusion modeling revealed that evidence accumulation was globally reduced for both stimulus types in lucid REM relative to wakefulness (words difference: -0.349, 95% HDI: [-0.660, -0.023]; pseudowords difference: -0.806, 95% HDI: [-1.072, -0.544], Fig 5B and Fig 5E) and N1 sleep (words difference: -0.372, 95% HDI: [-0.754, -0.014]; pseudowords difference: -0.341, 95% HDI: [-0.635, -0.056], Fig 5B and Fig 5E). Concurrently, participants adopted elevated decision thresholds, which increased reliably compared to wakefulness (words difference: 0.684, 95% HDI: [0.259, 1.149]; pseudowords difference: 0.453, 95% HDI: [0.102, 0.775], Fig 5C and 5F) and N1 sleep (words difference: 0.578, 95% HDI: [0.067, 1.075]; pseudowords difference: 0.599, 95% HDI: [0.261, 0.949], Fig 5C and 5F), with all credible intervals excluding zero. This threshold elevation, occurring alongside reduced evidence accumulation, contributed to relative preservation of word accuracy: by requiring more evidence before responding, participants maintained performance at 77% (vs. 86% in wake) despite impaired processing efficiency, while accepting substantially slower responses. Sensory-motor processing remained stable across states (all non-decision time 95% HDIs overlapped with 0, Fig 5A and 5D).

Finally, to determine if the threshold elevation in lucid REM was a direct compensation for drift reduction at the individual level, we analyzed the within-subject correlation between these parameter changes. We found no significant correlation (Pearson r = 0.268, p = 0.177). This suggests that while both adaptations occur reliably at the state level, they represent parallel strategic adjustments rather than a tightly coupled homeostatic mechanism.

These cross-state comparisons reveal two distinct patterns of cognitive adaptation during sleep. During N1 sleep, lexical decisions for words remained stable while pseudoword processing was selectively impaired, driven by reduced evidence accumulation and delayed sensory-motor processing. In contrast, lucid REM sleep showed global slowing and reduced evidence accumulation for both stimulus types, with compensatory threshold elevation that partially preserved word accuracy despite compromised processing efficiency.

### Lexical decision-making is absent in deeper sleep states

In contrast to N1 and lucid REM sleep, participants showed no evidence of lexical decision-making during N2 or non-lucid REM sleep. There were no significant differences in reaction times, accuracy, or any HDDM parameters between words and pseudowords (all 95% HDIs overlapped with 0, S3 Fig), indicating that complex decision-making does not occur under the neurophysiological conditions present in deeper sleep stages. However, given the lower trial counts in these stages, these results provide insufficient evidence to distinguish between a genuine absence of effects and limited statistical power.

These findings delineate clear boundaries for cognitive capacity during sleep: while N1 and lucid REM sleep support lexical decision-making through distinct, adaptive strategies, deeper sleep stages preclude such complex cognitive operations.

### Validation of the computational framework

We implemented a rigorous multi-step validation to ensure the reliability of our modeling results. First, we evaluated whether the complexity of our HDDM specification was warranted using Deviance Information Criterion (DIC) model comparison. The full model—in which drift rate, decision threshold, and non-decision time all varied by stimulus type, sleep stage, and their interaction—yielded a markedly lower DIC than three nested alternatives (no interactions, constant threshold, or constant non-decision time). The improvement in fit was substantial (ΔDIC = 40–67; S5 Table), indicating that the added complexity captured systematic condition-specific structure rather than overfitting noise [30].

Second, we conducted systematic parameter recovery analyses (N = 30 iterations) for the narcolepsy group. Recovery was excellent for the key parameters underpinning our conclusions: drift rate and non-decision time. Across group-level nodes, drift rate showed Pearson *r* = 0.990, mean |bias| = 0.042, and mean RMSE = 0.097; non-decision time showed Pearson *r* = 0.988, mean |bias| = 0.014, and mean RMSE = 0.040. Crucially, stimulus type contrasts (words vs. pseudowords) for both parameters were recovered with high precision (RMSE < 0.07). Decision thresholds exhibited good overall recovery but were numerically noisier in the lucid REM condition (RMSE = 0.229; mean |bias| = 0.195). Nevertheless, the lucid REM threshold elevation was consistently recovered in the same direction across all iterations (S4 Fig and S3–S4 Tables).

Third, to rule out the possibility that the threshold elevation in lucid REM sleep was a fitting artifact driven by parameter trade-offs, we compared the empirical effect to spurious effects generated from a null model (where thresholds were fixed). Although the null model yielded a mean spurious effect of 0.167 (SD = 0.124), the empirical lucid REM threshold effect (0.458) was substantially larger (approximately 2.7 × the mean spurious effect) and surpassed the maximum spurious value observed across all 30 simulations (0.346). This confirms that the observed threshold increase reflects a genuine strategic adjustment beyond fitting artifacts (S5 Fig).

Finally, we further assessed model fit using posterior predictive checks. The model reproduced the overall mean accuracy with high precision (observed: 81.0%; posterior predictive mean: 83.6%). For reaction times, the posterior predictive distributions showed reasonable agreement with the empirical data (observed median: 1.58 s; posterior predictive median: 1.26 s), capturing the central tendency and right-skew of the RT distributions, with a slight underestimation of the extreme upper tail (S6 Fig).

## Discussion

This study demonstrates that the capacity for lexical decision-making during sleep is not uniformly diminished, but instead shows distinct patterns of alteration across different states of consciousness. By employing drift diffusion modeling to analyze real-time behavioral responses in wakefulness, N1 sleep, and lucid REM sleep—across both healthy participants and participants with narcolepsy—we reveal that the cognitive mechanisms supporting decision-making are flexibly reorganized according to sleep state. These results provide direct evidence that sleep does not represent a globally inactive or disconnected condition. Rather, in line with previous findings [1,2], our results indicate that sleep involves dynamic, state-dependent reorganization of decision-making mechanisms. This adaptive reorganization allows some sleep stages to maintain or flexibly adjust specific cognitive functions, such as lexical decision-making, while other stages substantially limit or eliminate these abilities. Because healthy participants contributed very few sleep trials, especially in N1 and N2 stages, our core mechanistic conclusions are drawn from the narcolepsy group, with healthy participants serving primarily as a behavioral validation cohort.

A notable aspect of our data is that the computational profile of lexical decision-making during wakefulness diverged from findings in visual paradigms. Whereas prior studies of visual word recognition reported advantages for words over pseudowords in both sensory-motor and evidence accumulation components [22,31,32], our auditory task revealed a word advantage only in sensory-motor efficiency, with no difference in evidence accumulation. This likely reflects modality-specific processing, as auditory word recognition relies on sequential phonological encoding [23,24], rather than the parallel evidence integration characteristic of visual tasks. In participants with narcolepsy, sensory-motor processing for words was preserved but evidence accumulation was reduced, suggesting that while automatic processing of familiar stimuli remains intact, orexinergic dysfunction may selectively impact higher-order decision integration [29,33]. These baseline findings provide important context for interpreting state-dependent adaptations in lexical decision-making during sleep.

Importantly, our results show that different states of consciousness engage distinct computational strategies even for the same cognitive task. In N1 sleep, participants in both groups retained the ability to efficiently process familiar words, benefiting from both rapid sensory-motor responses and effective evidence accumulation. In contrast, lucid REM sleep was characterized by a reliance on post-sensory decision processing: evidence accumulation. Notably, these state-dependent patterns suggest that the brain does not simply become less efficient during sleep, but actively reorganizes how decisions are made depending on the underlying neural dynamics [18,34]. This computational reorganization likely reflects state-specific neural network configurations [15,16]. Specifically, N1 sleep preserves sufficient thalamocortical connectivity to support integrated sensory-cognitive processing [2,3,14], enabling both perceptual and decisional advantages for familiar words. In contrast, lucid REM sleep is characterized by altered neural dynamics—such as heightened prefrontal activity and a reconfigured sensory landscape [20,21,35] —which may contribute to the reduced sensory-motor efficiency and increased reliance on evidence accumulation observed in this state.

This dissociation—preserved lexical decisions for familiar words but impaired decisions for pseudowords during N1 sleep—demonstrates that automatic, well-practiced cognitive functions are less susceptible to the effects of light sleep than more demanding, controlled processes. Such a pattern aligns with the concept of hierarchical vulnerability among cognitive processes, wherein increasing task complexity or novelty confers greater sensitivity to sleep-related neural changes [2,3,14]. Specifically, automatic recognition of familiar words appears robust to the reduced thalamocortical connectivity and altered neural dynamics of N1 sleep, whereas controlled operations—such as evaluating novel pseudowords—are preferentially compromised. These findings argue against a model of uniform cognitive degradation during sleep, instead supporting the view that sleep selectively preserves access to well-consolidated and meaningful information, while constraining the engagement of resource-intensive, less automatized computations [17,36]. Such selective preservation may reflect an adaptive strategy, enabling the brain to maintain responsiveness to evolutionarily and experientially significant stimuli—even in states of diminished arousal or connectivity.

Lucid REM sleep, in contrast to both wakefulness and N1 sleep, reveals a distinctive pattern of decision-making adaptation. While sensory-motor processing remains robust across all three states, lucid REM sleep is marked by a global reduction in evidence accumulation for both words and pseudowords, reflecting a generalized decrease in the efficiency of information integration during lexical decision-making. Concurrent with this reduction, the brain adopts a more conservative decision policy by increasing the decision threshold—requiring more evidence before committing to a choice and thereby prioritizing accuracy over speed. This adaptive adjustment is consistent with the enhanced self-monitoring and volitional control characteristic of lucid dreaming [20,21], and demonstrates that, even in the face of altered neural dynamics, individuals retain the capacity to monitor and flexibly adapt their decision strategies [11,12]. Crucially, however, the lack of correlation between drift reduction and threshold elevation at the individual level suggests these are parallel state-dependent adaptations rather than a tightly coupled compensatory mechanism. Notably, these findings indicate that lucid REM sleep does not cause a uniform degradation of cognitive functions; rather, it selectively impacts the processes involved in gathering and evaluating information for decisions, while leaving sensory-motor pathways intact. This dissociation underscores that the integration of evidence and the adjustment of decision boundaries are particularly sensitive to sleep state, whereas perceptual and motor functions exhibit remarkable resilience.

The absence of lexical decision-making in N2 and non-lucid REM sleep clearly delineates the boundaries of cognitive capacity during sleep [37]. The lack of behavioral and computational differences between words and pseudowords in these states indicates that the brain is no longer able to selectively process or distinguish novel from familiar linguistic information. This finding supports the view that conscious, goal-directed cognition requires both thalamocortical connectivity and prefrontal engagement—features that are diminished or absent during these deeper sleep stages [25,38–41]. Notably, our modeling-based results contrast with those of [6], who reported above-chance behavioral responses to verbal stimuli in all sleep states using the same dataset. This discrepancy underscores a crucial distinction: while above-chance responses in deeper sleep may result from automatic motor activity or priming, our drift diffusion modeling reveals that these responses lack the computational hallmarks of genuine lexical decision-making—namely, differential evidence accumulation or threshold adjustment [11,22,32]. Thus, computational modeling offers a more nuanced perspective on cognitive functioning during sleep, effectively distinguishing complex, conscious decisions from automatic, stimulus-driven responses.

We acknowledge that altered states of consciousness could, in principle, engage decision mechanisms that deviate from standard accumulation-to-bound dynamics—for example, fragmented or intermittent integration and the use of simple heuristics [10,36]. In this study, we therefore treat the drift diffusion model as a principled computational framework for summarizing lexical decisions in terms of drift rate, threshold, and non-decision time, rather than as a literal description of all underlying neural computations during sleep. Our model comparison, parameter recovery, and posterior predictive analyses (S4–S6 Figs and S5 Table) indicate that this HDDM provides an adequate first-order description of the behavioral data, allowing relative changes in these parameters across states to be interpreted as meaningful indices of state-dependent reconfiguration. At the same time, more elaborate frameworks—for example, sequential sampling variants with intermittent or leaky accumulation, or models that explicitly formalize heuristic decision rules [11,42]—may capture additional aspects of sleep state decision dynamics, and we highlight systematic comparisons with such models as an important direction for future work.

Furthermore, our simulation-based validation analyses support the robustness of our computational inferences. Parameter recovery results indicate that the core DDM parameters—particularly drift rate and non-decision time—are identifiable under the present design. This provides sufficient sensitivity to detect the magnitude of word–pseudoword differences we report and to interpret the absence of threshold differences in N1 as a genuine null effect rather than a consequence of insufficient statistical power. Complementary null model simulations further suggest that the threshold increase observed in lucid REM reflects a genuine state-dependent adjustment rather than a fitting artifact. Together, these checks strengthen the conclusion that the drift diffusion framework provides a reliable characterization of decision-making reconfiguration across sleep states.

Our study has limitations that should guide future research. First, our lucid REM findings stem exclusively from participants with narcolepsy. While this population was necessary to obtain sufficient trials for modeling, future studies should extend these investigations to healthy participants—potentially using induction training protocols to increase lucid dream frequency—to test generalizability [43]. Second, although we rigorously excluded micro-arousals and confirmed sleep depth via spectral analysis (Türker et al., 2023) [6], the potential influence of brief, sub-threshold arousals remains a limitation. Crucially, however, the specific computational dissociation observed in N1—preserved processing for familiar words versus selective impairment for pseudowords—is inconsistent with generic wake contamination. If performance were driven by undetected wakefulness, we would expect a global facilitation across both stimulus types rather than the stimulus-specific dissociation we observed. Third, lucid REM was identified from the conjunction of an EMG-based lucidity code and post-nap subjective reports, and we adopted a pragmatic session-level rule whereby all REM epochs from naps with convergent EMG and subjective evidence were labeled as lucid REM. This approach likely included some non-lucid REM epochs and thus may have biased our estimates toward those of non-lucid REM, making the reported lucid REM effects conservative; future work with denser lucidity signaling (e.g., repeated motor or oculomotor codes) and larger datasets will be needed to separate lucid and non-lucid REM at finer temporal resolution and to refine the computational characterization of decision-making across these REM substates.

In conclusion, our findings reveal a landscape of dynamic reorganization where the brain selectively preserves access to meaningful information and adapts decision strategies to match the available neural resources. This perspective advances our understanding of the computational mechanisms supporting cognitive flexibility and consciousness, highlighting the adaptive potential of altered neural states.

## Methods

### Ethics statement

The study adhered to the Declaration of Helsinki, and ethical approval was granted by the local ethics committee (Comité de Protection des Personnes [CPP] Ile-de-France 8). All participants provided written informed consent before participation.

This study presents a novel analysis of data originally collected in 2020 at the Sleep Clinic of Pitié-Salpêtrière Hospital, France, as part of an experiment previously published [6].

Thirty individuals diagnosed with narcolepsy (NP; 14 women; mean age: 35 ± 11 years) and 22 healthy participants (HP; 10 women; mean age: 24 ± 4 years) were recruited. Participants with narcolepsy were diagnosed according to international diagnostic criteria and recruited from the National Reference Center for Narcolepsy at the Pitié-Salpêtrière Hospital. Among NPs, 80% reported frequent lucid dreaming (≥3 lucid dreams per week), whereas none of the HPs reported a history of lucid dreaming. Three participants (two NPs and one HP) were excluded due to technical issues during data acquisition, leaving 27 NPs (21 frequent lucid dreamers) and 21 HPs in the final analyses. Participants were compensated financially for their involvement. For detailed demographic and clinical information, see the previously published study [6].

### Experimental design

#### Task overview.

Participants performed a lexical decision task in which they determined whether auditory stimuli were real words or pseudowords. Responses were indicated via brief contractions of facial muscles: the corrugator (frowning) and zygomatic (smiling) muscles. The muscle-response mappings were counterbalanced across participants. Stimuli were presented in pseudorandomized order, ensuring each stimulus was presented only once to prevent repetition effects. Participants completed a 10-minute familiarization session before data collection to practice the task and ensure comfort with the auditory stimuli, which were played at an average volume of 48 dB and adjusted for individual audibility.

#### Nap protocol.

Participants with narcolepsy completed five 20-minute nap sessions, interspersed with 80-minute breaks, while HPs completed a single uninterrupted 100-minute daytime nap. Each nap session consisted of 10 active (“ON”) periods, during which six stimuli (three words, three pseudowords) were presented every 9–11 seconds against a background of continuous white noise. These “ON” periods alternated with 1-minute “OFF” intervals, during which only white noise was delivered. Across sessions, 60 stimuli (30 words, 30 pseudowords) were presented per participant, with presentation lists randomized to mitigate order effects.

#### Stimuli.

Auditory stimuli were selected from the MEGALEX database [44] and included French words and pseudowords spoken by a female voice. Stimuli were standardized to a duration of 690 ms and controlled for frequency and emotional valence. To ensure consistency, each participant received five unique stimulus lists, randomized across nap sessions. Stimuli were delivered via speakers using Psychtoolbox in MATLAB (MathWorks), with a randomized inter-stimulus interval of 9–11 seconds.

#### Electrophysiological recording.

Electrophysiological data were collected using a 10-channel EEG setup (Fp1, Fp2, Cz, C3, C4, Pz, P3, P4, O1, O2), following the international 10–20 system. Signals were referenced to the right mastoid (A2 electrode). Additional recordings included electrooculography (EOG) from two electrodes positioned to capture eye movements, electromyography (EMG) from three channels (chin muscles for sleep staging and the zygomatic and corrugator muscles to record behavioral responses), and electrocardiography (ECG) from one channel to record heart activity. All signals were recorded continuously at a sampling rate of 2,048 Hz using a Grael 4K PSG/EEG amplifier (Medical Data Technology, Compumedics).

#### Sleep scoring and identification of lucid dream.

Sleep stages were scored offline by a certified sleep expert according to American Academy of Sleep Medicine guidelines [45] using Profusion (Compumedics). EEG and EOG signals were filtered between 0.3–15 Hz, EMG between 10–100 Hz, and ECG between 0.3–70 Hz. Sleep stages were scored in 30-second epochs as wakefulness, N1, N2, N3, or REM sleep. Micro-arousals were defined as alpha activity lasting 3–15 seconds, with arousals exceeding 15 seconds classified as wakefulness. For REM sleep, micro-arousals were further characterized by transient increases in EMG tone. Trials containing micro-arousals were excluded from subsequent analyses.

Lucid REM sleep was identified using a combination of online EMG-based lucidity signals and post-nap subjective reports. During the instruction phase, participants were asked to signal the onset of a lucid dream by producing a predefined “mixed code” of facial muscle activity—one corrugator (frowning) contraction followed by one zygomatic (smiling) contraction—recorded on the same EMG channels used for lexical decision responses. These lucidity signals were monitored online during the nap sessions and verified offline, and they showed strong concordance with participants’ reports upon awakening. For naps in which both the EMG-based lucidity signal and the post-nap report indicated a lucid dream, all REM epochs from that session were classified as lucid REM sleep for the purposes of the main analyses. We adopted this session-level classification rule to ensure sufficient trial numbers per condition for hierarchical drift diffusion modeling, acknowledging that this approach may include a small proportion of non-lucid REM within the lucid REM category and therefore yields conservative estimates of lucid REM effects. No healthy participants reported lucid dreams.

#### Muscle response analysis.

EMG signals were segmented into 10-second mini-epochs corresponding to sleep stages. Mini-epochs with micro-arousals were excluded. Muscle contractions were classified as valid responses if at least two consecutive contractions were detected; single contractions (twitches) were excluded as non-responses. Scoring reliability was validated by reanalyzing 10% of the data with a second blinded scorer, yielding 84% agreement.

#### Drift diffusion model analysis.

To investigate the mechanisms underlying lexical decision-making across wakefulness and sleep stages, we employed the Drift Diffusion Model (DDM), a well-established framework for modeling two-choice decision-making tasks [11]. The DDM assumes that decisions arise from a continuous process of evidence accumulation, where sensory information about the two options (e.g., words and pseudowords) is integrated over time until a decision threshold is reached. The model decomposes behavioral data (accuracy and response times) into distinct cognitive parameters, providing insights into the underlying decision-making processes. The DDM decomposes this process into four main components: the starting point (***z***), which indicates a predecision bias; the nondecision time (***t***), which covers factors unrelated to the actual decision and is often linked to the encoding, motor execution, and lexical access in lexical tasks; the drift rate (***v***), which represents the speed of information accumulation; and the decision threshold (***a***), which indicate when enough evidence has been collected to make a decision.

Previous findings suggest that differences in lexical decision behavior are primarily driven by drift rate and non-decision time [22,31]. However, to account for potential variations in decision-making strategies across wakefulness and sleep, we also included decision threshold (***a***) in our model. This approach allowed us to examine how drift rate (***v***), non-decision time (***t***), and decision threshold (***a***) were modulated by word type and state of consciousness (wakefulness and sleep states). Predecision bias (starting point) was estimated at the participant level and assumed to remain constant across word type and sleep states, as our primary interest lay in the interaction between evidence accumulation, decision thresholds, and state-dependent cognitive dynamics [22,31,46]. Separate HDDM analyses were conducted for HP and NP to assess group-specific effects (Fig 1C).

We used a Bayesian hierarchical approach with the HDDM 0.8 tool in Docker to estimate these parameters [47], assuming that participants’ parameters are drawn from a shared distribution. We applied Markov Chain Monte Carlo (MCMC) sampling to generate 10,000 samples, discarding the first 1,000 as burn-in. Model convergence was checked by inspecting trace plots, autocorrelation, and the Gelman–Rubin R-hat statistic (ensuring R-hat < 1.1). We applied HDDM regression analysis separately for HP and NP using the following model:

$a,v,t=\;\beta_{\text{0}j}+\beta_{\text{1}j}*Word_{type}+\beta_{\text{2}j}*Sleep_{stages}+\beta_{\text{3}j}*Word_{type}*Sleep_{stages}$


$$\;z=\;\beta_{\text{0}j}$$

#### Parameter recovery analysis.

To evaluate the reliability and identifiability of our hierarchical drift diffusion model (HDDM) parameter estimates, we conducted systematic parameter recovery analyses following established recommendations for simulation-based model validation [13,48]. Parameter recovery was performed for the narcolepsy (NP) group only, because healthy participants contributed substantially fewer trials during sleep stages (on average 3–5 trials per condition in N1/N2; S1 Table), which precluded reliable convergence for complex hierarchical recovery. In contrast, NP participants provided sufficient trials across wakefulness and all sleep stages to support robust estimation (S2 Table).

For the NP group, we conducted 30 independent recovery iterations. In each iteration, we followed a three-step procedure. First, we simulated a synthetic dataset by generating behavioral responses (accuracy and reaction times) from the fitted hierarchical model, using the group-level posterior means as the “true” generating values. The simulated data preserved the exact trial structure of the empirical dataset, including the number of trials per participant, stimulus type (words vs. pseudowords), and sleep stage (wake, N1, N2, REM, lucid REM). Data generation was implemented with the built-in generative functions in HDDM (version 0.8.0; Wiecki et al., 2013). Second, we re-fitted the identical HDDM regression specification to each simulated dataset, using the same priors, hierarchical structure, and MCMC settings as in the main analysis (one chain with 15,000 samples, discarding the first 2,000 samples as burn-in). At the trial level, drift rate (***v***), decision threshold (***a***), and non-decision time (***t***) were modeled as linear functions of stimulus type, sleep stage, and their interaction. Third, after fitting each recovery model, we extracted the group-level posterior means for all regression coefficients and compared the recovered parameters with the true values used for data simulation.

Recovery quality was quantified using three complementary metrics computed across all group-level nodes and iterations. First, we calculated the Pearson correlation (r) between true and recovered parameter values, indexing overall fidelity of estimation. Second, we quantified bias as the mean difference between recovered and true values (recovered − true) for each node, averaged across iterations; values close to zero indicate minimal systematic over- or under-estimation. Third, we computed the root mean square error (RMSE), defined as the square root of the mean squared difference between recovered and true values, capturing both systematic bias and random variability. For descriptive purposes, and in line with common practice in parameter-recovery studies in cognitive modeling (e.g., [13,48]), we classified recovery as “excellent” when r > 0.90, |bias| < 0.05, and RMSE < 0.10; “good” when r > 0.80, |bias| < 0.10, and RMSE < 0.20; and “acceptable” when r > 0.70, |bias| < 0.15, and RMSE < 0.30. These ranges were defined a priori as pragmatic benchmarks to summarize recovery quality rather than as strict cut-offs, and parameters that failed to meet at least the “acceptable” criterion were flagged as requiring more cautious interpretation in the Results and Discussion.

#### Null model simulation to test parameter trade-offs.

To verify that the observed threshold elevation in lucid REM sleep reflected a genuine strategic adjustment rather than an artifact of parameter trade-offs, we conducted null model simulations. We generated 30 synthetic datasets in which drift rates varied across conditions (based on empirical posterior means), but decision thresholds were fixed at the grand mean (a = 2.54) for all conditions. This null model thus assumes no true differences in decision threshold across sleep stages or stimulus types. For each synthetic dataset, we then fitted the full HDDM regression model used in the main analysis, with thresholds, drift rates, and non-decision times allowed to vary by stimulus type, sleep stage, and their interaction (15,000 MCMC samples, first 2,000 discarded as burn-in). We extracted the lucid REM-related threshold regression coefficient from each fit and treated deviations from zero as spurious effects arising solely from parameter trade-offs under a true null for threshold. The distribution of these spurious effects was then compared to the empirical lucid REM threshold effect to assess whether the latter could plausibly be explained by fitting artifacts.

#### Posterior predictive checks.

To assess the descriptive accuracy of our HDDM, we performed posterior predictive checks. Using the fitted group-level posterior parameter estimates as generating values, we simulated 30 synthetic datasets that preserved the exact empirical trial structure (number of trials per participant, stimulus type, and sleep stage). For each simulated dataset, we computed the distributions of accuracy and reaction times and compared them to the corresponding empirical distributions. This allowed us to evaluate whether the model reproduced key behavioral patterns, including accuracy differences across sleep stages and the characteristic shape of the reaction time distributions.

#### Within-subject correlation analysis.

To test whether the threshold elevation in lucid REM sleep was mechanistically coupled to drift rate reduction at the individual level, we performed a within-subject correlation analysis. We extracted individual-level parameter estimates (drift rate ***v*** and threshold ***a***) for wakefulness and lucid REM sleep. We calculated the change in each parameter (Lucid REM – Wake) for every participant and computed the Pearson correlation between the change in drift rate and the change in threshold.

#### Model comparison.

To evaluate whether the complexity of our HDDM specification was warranted, we conducted formal model comparisons using the Deviance Information Criterion (DIC). For the narcolepsy (NP) group, we fitted four hierarchical drift–diffusion models that differed only in which parameters were allowed to vary across conditions, while keeping priors, hierarchical structure, and sampling settings constant (10,000 MCMC samples, first 2,000 discarded as burn-in). The full model allowed drift rate (***v***), decision threshold (***a***), and non-decision time (***t***) to vary as linear functions of stimulus type (words vs. pseudowords), sleep stage, and their interaction (stimulus × stage). We compared this full model against three nested alternatives: (1) a main-effects model in which ***v***, ***a***, and ***t*** varied additively with stimulus type and sleep stage but without interaction terms; (2) a constant-threshold model in which ***a*** was held fixed across conditions, while ***v*** and ***t*** varied by stimulus × stage; and (3) a constant non-decision time model in which ***t*** was held fixed, while ***v*** and ***a*** varied by stimulus × stage. DIC quantifies out-of-sample predictive adequacy while penalizing model complexity; lower DIC values indicate a better trade-off between fit and parsimony. Differences in DIC (ΔDIC) greater than 10 are conventionally interpreted as strong evidence in favor of the model with the lower DIC [30].

Given the sparse number of sleep trials contributed by healthy participants, particularly in N1 and N2 sleep (S1 Table), our primary hierarchical drift diffusion modeling and mechanistic inferences focus on the narcolepsy group. Healthy participants are included with a more restricted and auxiliary aim: to provide a behavioral validation of the task and analysis pipeline, by testing whether wake and light sleep (N1/N2) performance patterns qualitatively align with prior literature and theoretical expectations.

### Statistics

We only included response trials where stimuli were presented in the current study. Additionally, trials with microarousal (HP: 15%; NP: 13.6%) were excluded from the analysis. N3 (0.07%) and REM (1%) sleep trials in HP were excluded due to insufficient trial numbers. To eliminate the possibility of random muscle contractions, we excluded trials in which participants exhibited only a single muscle contraction (HP: 0.6%; NP: 1.5%). Finally, we excluded trials with reaction times (RT) shorter than 0.69 s or longer than 9.9 s. Additionally, we removed outliers using a conservative criterion of mean ± 2.5 median absolute deviation (MAD). In the healthy participant group, these criteria resulted in the exclusion of 3 trials (0.51%) based on RT bounds and 66 trials (11.26%) based on outlier status. In the narcolepsy group, 28 trials (2.04%) were excluded due to RT bounds and 157 trials (11.44%) as outliers.

Our study aimed to uncover the computational processes underlying lexical decision-making across wakefulness and sleep in healthy participants (HP) and individuals with narcolepsy (NP) using the drift diffusion framework. Participants performed a lexical decision task (LDT) during wakefulness and continued the task throughout sleep, using facial muscle contractions (zygomatic and corrugator muscles) to indicate whether spoken stimuli were words or pseudowords in the French lexicon (Fig 1A). Polysomnography, along with additional EMG sensors, was used to confirm sleep stages and capture behavioral responses. This setup allowed us to collect both accuracy and RT data for lexical judgments during wakefulness and sleep. To analyze behavioral performance, we applied a Bayesian linear mixed model (BLMM) to trial-level accuracy and RT data. Fixed factors included word type (words vs. pseudowords) and sleep stages, while participants were modeled as a random factor. BLMM is particularly suited for analyzing hierarchical data structures, addressing individual variability and imbalances in trial numbers [49–51].

The model is specified as follows:


$$\mu_{j}=\;\beta_{\text{0}j}+\beta_{\text{1}j}*Word_{type}+\beta_{\text{2}j}*Sleep_{stages}+\beta_{\text{3}j}*Word_{type}*Sleep_{stages}$$ (1)

Where $\mu_{j}$ represents either response accuracy or reaction time, and j represents the subject. For response accuracy, we used the Bernoulli family to model the binary data. For reaction time, we employed the shifted lognormal family to appropriately model the RT data.

During the analysis of reaction time, we conducted a control analysis by adding response accuracy as a fixed factor to examine whether correct or incorrect responses would show different reaction times:


$$\mu_{j}=\;\beta_{\text{0}j}+\beta_{\text{1}j}*Word_{types}+\beta_{\text{2}j}*Accuracy+\beta_{\text{3}j}*Sleep_{stages}+\beta_{\text{4}j}*Word_{type}*Accuracy*Sleep_{stages}$$ (2)

For each model, we ran four MCMC chains with 5,000 samples each, discarding the first 500 samples as a warm-up. We assessed model convergence using the Gelman–Rubin R-hat statistic, ensuring (R-hat < 1.1). Statistical inferences were based on the 95% Highest Density Interval (HDI) of the posterior distribution. Effects were considered significant if the 95% HDI did not include 0.

## Supporting information

S1 Table Mean and SEM of post-exclusion trial numbers across sleep states and word types in the Healthy Participant (HP) group. (DOCX)

S2 Table Mean and SEM of post-exclusion trial numbers across sleep states and word types in the Narcolepsy (NP) group. (DOCX)

S3 Table Parameter recovery metrics by parameter type. Note: Quality ratings based on established thresholds (Ratcliff & Childers, 2015): Excellent: |bias| < 0.05, RMSE < 0.10; Good: |bias| < 0.10, RMSE < 0.20.
(DOCX)

S4 Table Recovery quality for key theoretical predictions. Interpretation: Bias < 0.05 indicates minimal systematic error; RMSE reflects absolute estimation precision. All key predictions show acceptable to excellent recovery except lucid REM threshold (RMSE = 0.229), which we now acknowledge as uncertain in magnitude but reliable in direction.
(DOCX)

S5 Table Model comparison results. *Note:* Lower DIC values indicate better model fit. ΔDIC represents the difference in DIC relative to the best-fitting (Full) model. By convention, ΔDIC > 10 indicates strong evidence against the model with the higher DIC. The Full Model provides the best fit, justifying the inclusion of all parameters and interaction terms.
(DOCX)

S1 Fig Reaction time differences between words and pseudowords conditioned on response accuracy. Posterior distributions of reaction times (RT) for Healthy Participants (HP) and Narcolepsy Patients (NP), separated into Correct and Incorrect responses. The X-axis denotes the estimated mean RT (in seconds). Blue lines represent Words and light blue lines represent Pseudowords (horizontal bars indicate the 95% Highest Density Interval, HDI). The purple line displays the posterior contrast (difference) between Words and Pseudowords. A difference is considered statistically credible if the 95% HDI of the contrast (purple) excludes zero (vertical gray line); overlap with zero indicates no reliable difference.
(EPS)

S2 Fig Behavioral and computational mechanisms of lexical decision during N1 sleep in healthy participants. (A) Behavioral results (Response Accuracy and Reaction Time) estimated via Bayesian Linear Mixed Models (BLMM). (B–D) Posterior distributions of HDDM parameters during N1 sleep: (B) Non-decision time (*t*); (C) Drift rate (*v*); and (D) Decision threshold (*a*). In all panels, blue lines represent Words and light blue lines represent Pseudowords (horizontal bars indicate the 95% Highest Density Interval, HDI). Asterisks (*) denote credible differences, defined as the 95% HDI of the contrast excluding zero (vertical gray line).
(EPS)

S3 Fig Absence of lexical decision-making signatures during N2 sleep and Non-Lucid REM sleep. Behavioral performance and computational parameters for N2 sleep (in both Healthy Participants and Participants with Narcolepsy) and Non-Lucid REM sleep (in Participants with Narcolepsy). (A, B, I) Behavioral results: Estimated mean response accuracy and reaction time. Blue lines represent Words and light blue lines represent Pseudowords. (C–H, J) HDDM Parameters: Posterior distributions for Non-decision time (*t*), Drift rate (*v*), and Decision threshold (*a*). Left sub-panels show parameter estimates; right sub-panels show the contrast (difference). In all panels, the 95% Highest Density Interval (HDI) of the contrast overlaps with zero (vertical gray line), indicating no reliable difference between words and pseudowords.
(EPS)

S4 Fig Parameter recovery validation for narcolepsy group HDDM Analysis. (A) Scatter plots comparing true parameter values (x-axis) used to simulate data versus recovered parameter values (y-axis) from re-fitting the HDDM model. Each point represents one parameter estimate from one of 30 recovery iterations. Dashed red line indicates perfect recovery (slope = 1, intercept = 0). Pearson correlations (r), root mean square errors (RMSE), and bias values are shown for each parameter type. (B) Distribution of recovery errors (recovered - true) across all nodes and iterations. Red dashed line indicates zero error (perfect recovery); orange solid line shows mean error (bias). Drift rate (*v*) and non-decision time (*t*) show near-zero bias with tight distributions, indicating excellent recovery. Decision threshold (*a*) shows wider distribution but centered near zero, indicating unbiased but noisier estimation.
(EPS)

S5 Fig Null model simulation validates Lucid REM threshold elevation. Distribution of spurious threshold effects for the lucid-REM parameter from 30 null-model simulations. Synthetic datasets were generated with constant decision thresholds (*a* = 2.54) but varying drift rates matching the empirical structure. The full hierarchical drift diffusion model was then fitted to each dataset, allowing thresholds to vary freely, to quantify spurious threshold effects arising purely from parameter trade-offs during fitting. Blue bars indicate spurious effects below the 95th percentile; coral bars indicate the upper 5% tail. The gray vertical line marks zero effect. The orange dashed line shows the mean spurious effect (0.167 ± 0.124 SD), and the orange dash-dotted line marks the 95th percentile (0.330). The dark red line indicates the empirical lucid REM threshold effect (0.458), which exceeds all 30 null iterations (maximum spurious = 0.346) and is approximately 2.7 times larger than the mean spurious effect. This provides strong evidence that the threshold elevation in lucid REM reflects a genuine state-dependent increase in response caution rather than a statistical artifact of model fitting.
(EPS)

S6 Fig Posterior predictive checks for reaction times. Posterior predictive assessment of model fit for reaction-time distributions in the narcolepsy group. The observed empirical density (black line) is overlaid with the posterior-predictive density (blue line), obtained by simulating datasets from the fitted hierarchical model using group-level posterior parameters. The orange dashed line indicates the posterior-predictive median. The model successfully captures the central tendency and characteristic right-skew of the reaction-time data, despite a modest underestimation of the extreme upper tail.
(EPS)
