# Supplementary material for: How sleeping minds decide: State-specific reconfigurations of lexical decision-making
Source: PLoS Comput Biol. 2026 Feb 23;22(2):e1014007. doi: 10.1371/journal.pcbi.1014007 (PMC12948133; doi:10.1371/journal.pcbi.1014007)
Supplement: S1 Table — (DOCX) [file pcbi.1014007.s001.docx]

**S1 Table. Mean and SEM of post-exclusion trial numbers across sleep states and word types in the Healthy Participant (HP) group.**

|  | Healthy Group | | |
| --- | --- | --- | --- |
|  | Wake (Mean ± SE) | N1(Mean ± SE) | N2(Mean ± SE) |
| Words | 48.2 ± 7.92 | 5 ± 0.89 | 3 ± 0.59 |
| Pseudowords | 46.7 ± 7.67 | 3.33 ± 0.44 | 2.77 ± 0.71 |
