# Supplementary material for: How sleeping minds decide: State-specific reconfigurations of lexical decision-making
Source: PLoS Comput Biol. 2026 Feb 23;22(2):e1014007. doi: 10.1371/journal.pcbi.1014007 (PMC12948133; doi:10.1371/journal.pcbi.1014007)
Supplement: S2 Table — (DOCX) [file pcbi.1014007.s002.docx]

**S2 Table. Mean and SEM of post-exclusion trial numbers across sleep states and word types in the Narcolepsy (NP) group.**

|  | Individual with narcolepsy | | | | |
| --- | --- | --- | --- | --- | --- |
|  | Wake  (Mean ± SE) | N1  (Mean ± SE) | N2  (Mean ± SE) | REM  (Mean ± SE) | Lucid REM  (Mean ± SE) |
| Words | 26.4 ± 5.79 | 9.04 ± 1.68 | 8.92 ± 9.17 | 12.2 ± 3.97 | 14.4 ± 2.15 |
| Pseudowords | 27.1 ± 5.94 | 8.69 ± 1.53 | 9.17 ± 1.78 | 11.6 ± 3.64 | 15.5 ± 2.24 |
