# Supplementary material for: How sleeping minds decide: State-specific reconfigurations of lexical decision-making
Source: PLoS Comput Biol. 2026 Feb 23;22(2):e1014007. doi: 10.1371/journal.pcbi.1014007 (PMC12948133; doi:10.1371/journal.pcbi.1014007)
Supplement: S3 Table — Note: Quality ratings based on established thresholds (Ratcliff & Childers, 2015): Excellent: |bias| < 0.05, RMSE < 0.10; Good: |bias| < 0.10, RMSE < 0.20. (DOCX) [file pcbi.1014007.s003.docx]

**S3 Table. Parameter recovery metrics by parameter type**

| Parameter | N Nodes | Mean \|Bias\| | Mean RMSE | Quality Rating |
| --- | --- | --- | --- | --- |
| v (drift) | 10 | 0.042 | 0.097 | Excellent |
| t (NDT) | 10 | 0.014 | 0.040 | Excellent |
| a (thresh) | 10 | 0.096 | 0.168 | Good |

Note: Quality ratings based on established thresholds (Ratcliff & Childers, 2015): Excellent: |bias| < 0.05, RMSE < 0.10; Good: |bias| < 0.10, RMSE < 0.20
