# Supplementary material for: How sleeping minds decide: State-specific reconfigurations of lexical decision-making
Source: PLoS Comput Biol. 2026 Feb 23;22(2):e1014007. doi: 10.1371/journal.pcbi.1014007 (PMC12948133; doi:10.1371/journal.pcbi.1014007)
Supplement: S4 Table — Interpretation: Bias < 0.05 indicates minimal systematic error; RMSE reflects absolute estimation precision. All key predictions show acceptable to excellent recovery except lucid REM threshold (RMSE = 0.229), which we now acknowledge as uncertain in magnitude but reliable in direction. (DOCX) [file pcbi.1014007.s004.docx]

**S4 Table. Recovery quality for key theoretical predictions**

| Node Description | Parameter | True Value | Mean Recov | Bias | RMSE |
| --- | --- | --- | --- | --- | --- |
| Stimulus: Words vs Pseudo | v | -0.186 | -0.212 | -0.026 | 0.066 |
| Stimulus: Words vs Pseudo | t | -0.101 | -0.098 | 0.003 | 0.019 |
| Stimulus: Words vs Pseudo | a | -0.185 | -0.228 | -0.043 | 0.101 |
| LucidREM: Threshold elevation | a | 0.458 | 0.262 | -0.195 | 0.229 |
| N1 Sleep: Drift reduction | v | -0.466 | -0.493 | -0.027 | 0.083 |

Interpretation: Bias < 0.05 indicates minimal systematic error; RMSE reflects absolute estimation precision. All key predictions show acceptable to excellent recovery except lucid REM threshold (RMSE = 0.229), which we now acknowledge as uncertain in magnitude but reliable in direction.
