# Supplementary material for: How sleeping minds decide: State-specific reconfigurations of lexical decision-making
Source: PLoS Comput Biol. 2026 Feb 23;22(2):e1014007. doi: 10.1371/journal.pcbi.1014007 (PMC12948133; doi:10.1371/journal.pcbi.1014007)
Supplement: S5 Table — Note: Lower DIC values indicate better model fit. ΔDIC represents the difference in DIC relative to the best-fitting (Full) model. By convention, ΔDIC > 10 indicates strong evidence against the model with the higher DIC. The Full Model provides the best fit, justifying the inclusion of all parameters and interaction terms. (DOCX) [file pcbi.1014007.s005.docx]

**S5 Table. Model comparison results**

| **Model Specification** | **Parameters Allowed to Vary** | **DIC** | **ΔDIC**  **(vs. Full Model)** |
| --- | --- | --- | --- |
| **Full Model** | *v, a, t* (Stimulus × Stage) | **9087.95** | **0** |
| No Interactions | *v, a, t* (Stimulus + Stage) | 9127.88 | +39.93 |
| Constant Threshold | *v, t* (Stimulus × Stage); *a* (fixed) | 9151.32 | +63.37 |
| Constant Non-decision Time | *v, a* (Stimulus × Stage); *t* (fixed) | 9155.07 | +67.13 |

*Note:* Lower DIC values indicate better model fit. **Δ**DIC represents the difference in DIC relative to the best-fitting (Full) model. By convention, **Δ**DIC > 10 indicates strong evidence against the model with the higher DIC. The Full Model provides the best fit, justifying the inclusion of all parameters and interaction terms.
